# Lossless hybridization between photovoltaic and thermoelectric devices

**DOI:** 10.1038/srep02123

**Published:** 2013-07-03

**Authors:** Kwang-Tae Park, Sun-Mi Shin, Abdullah S. Tazebay, Han-Don Um, Jin-Young Jung, Sang-Won Jee, Min-Wook Oh, Su-Dong Park, Bongyoung Yoo, Choongho Yu, Jung-Ho Lee

**Affiliations:** 1 Department of Chemical Engineering, Hanyang University, Ansan, 426-791, Korea; 2 Creative Electrotechnology Research Center, Korea Electrotechnology Research Institute (KERI), Changwon, 642-120, Korea; 3 Department of Materials Engineering, Hanyang University, Ansan, 426-791, Korea; 4 Department of Mechanical Engineering, Texas A&M University, College Station, Texas 77843, United States; 5 These authors contributed equally to this work.

## Abstract

The optimal hybridization of photovoltaic (PV) and thermoelectric (TE) devices has long been considered ideal for the efficient harnessing solar energy. Our hybrid approach uses full spectrum solar energy *via* lossless coupling between PV and TE devices while collecting waste energy from thermalization and transmission losses from PV devices. Achieving lossless coupling makes the power output from the hybrid device equal to the sum of the maximum power outputs produced separately from individual PV and TE devices. TE devices need to have low internal resistances enough to convey photo-generated currents without sacrificing the PV fill factor. Concomitantly, a large number of p-n legs are preferred to drive a high Seebeck voltage in TE. Our simple method of attaching a TE device to a PV device has greatly improved the conversion efficiency and power output of the PV device (~30% at a 15°C temperature gradient across a TE device).

Enhancing the utilization of solar energy and waste heat recovery, in order to mitigate the global energy crisis, are in demand because these energy sources are readily accessible and abundant, in contrast to wind, water, and pressure^1^,^2^,^3^,^4^,^5^,^6^,^7^,^8^,^9^,^10^,^11^,^12^,^13^,^14^,^15^,^16^,^17^,^18^. Photovoltaic (PV)^2^,^3^,^4^,^5^,^6^,^7^,^8^,^9^,^10^ and thermoelectric (TE)^13^,^14^,^15^,^16^,^17^,^18^ devices have therefore been studied to increase cell conversion efficiency and the thermoelectric figure of merit, respectively. However, their conversion performance still does not meet industrial requirements. One promising approach to further improve conversion efficiency is to combine PV and TE devices. This would allow harvesting of a larger spectrum of solar energy along with the waste heat generated from the solar facing PV^19^,^20^,^21^,^22^,^23^,^24^,^25^. In PV operation, ~40% of solar spectral irradiance is spontaneously transformed into heat by both thermalization loss of high energy photons and transmission loss of low energy photons^26^. Therefore, additional energy harvesting from waste heat is useful not only for increasing the efficiency but also for removing unwanted heat that prevents efficient PV operation^27^.

A few recent reports have shown a technological progress toward the PV-TE hybrid methodology. For example, a general strategy of PV-TE hybrid devices was suggested using a spectrum splitter to partition a broad solar spectrum into PV and TE^20^. Yang *et al.* also designed a hybrid system using water tubes to serve as a heat sink, allowing heat to be easily transferred into flowing water^23^. The hybrid cell consisting of a dye-sensitized solar cell and a TE device was also reported since sensitizers are limited to absorb low energy photons whose wavelengths are longer than 600 nm^25^. The focus was to improve the interfacial contact between PV and TE components to effectively heat the hot side of the TE device. However, lossless matching for optimized hybrid operation of the two different circuits, which is critical for efficiency optimization, has not been studied. For instance, unoptimized crosstalks in combined circuits often increase total series resistance, which destroys the synergistic effects expected from circuit hybridization. In particular, matching the internal resistance of TE devices with PV circuits for lossless coupling is critical. Here, we demonstrate the PV-TE hybrid device optimized to realize the lossless coupling between PV and TE devices. In addition, a semi-quantitative theoretical approach has been presented for understanding the lossless hybridization.

At the initial stage of hybrid current–voltage (*I*–*V*) operation, the temperature gradient across the TE device was zero (ΔT = 0°C). Hence, the maximum power output was lower than the sole PV output because the fill factor (*FF*) of the hybrid circuit decreased due to the internal resistance of the TE device without additional voltage gains. As heat was supplied to the TE part, the output voltage in the hybrid circuit increased due to the Seebeck effect. Upon optimizing the hybrid circuit (lossless coupling), we observed a remarkable improvement in the PV device: its efficiency increased by ~30% (conversion efficiency of 16.3%) with only a 15°C temperature gradient across the TE device.

## Results

**Figure 1:**
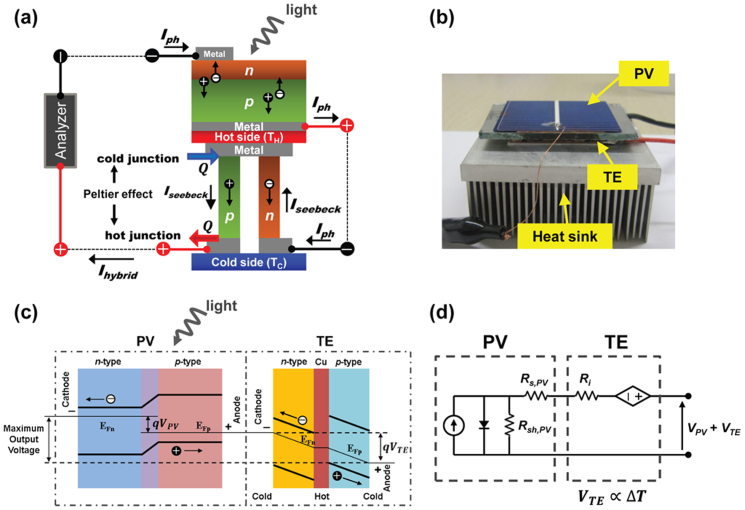
PV-TE hybrid device. (a) Schematic illustration of a hybrid circuit consisting of a photovoltaic (PV) and a thermoelectric (TE), which are placed in tandem and electrically connected in series. The abbreviations are: photo-generated current (*I_ph_*), Seebeck current (*I_seebeck_*), and output current in the hybrid circuit (*I_Hybrid_*). (b) Optical image of prototype PV-TE hybrid circuit. (c) Electron energy band diagram of the PV-TE hybrid circuit showing the carrier transfer process and output voltage under light illumination and thermal gradient. (d) The corresponding equivalent electric circuit diagram. The PV cell is regarded as a current source shunted by a diode with a series resistance (*R_s,PV_*) and a shunt resistance (*R_sh,PV_*). The TE module is simply represented by a voltage source connecting with an internal series resistance (*R_i_*).

Our hybrid power generation system consisted of a crystalline Si PV and a bismuth-tellurium based commercial TE device, which were placed in tandem, and electrically connected in series (Figs. 1a and b). The PV devices used in our hybrid circuits were conventional crystalline Si solar cells with an open-circuit voltage (*V_oc_*) of 0.592 V, a short-circuit current density (*J_sc_*) of 30 mA/cm^2^, and a conversion efficiency of 12.5% under AM 1.5 G normal illumination of 100 mW/cm^2^ at room temperature (25°C). This PV device was purposely chosen because its output current was comparable to that of the TE device. In order to investigate PV-TE coupling conditions without power loss, three different TE devices were used:

1. T21S: the number (*N*) of *p*-*n* couples was 127, small size (4 cm^2^), and internal resistance (*R_i_*) of TE was 2.1 Ω,
2. T12S: *N* = 31, small size (4 cm^2^), *R_i_* = 1.2 Ω,
3. T19L: *N* = 127, large size (16 cm^2^), *R_i_* = 1.9 Ω.

The *I*–*V* characteristics of the PV, TE, and hybrid circuits were experimentally investigated in order to evaluate the power loss upon the PV-TE hybridization. Analytical results for hybrid operations under various conditions are also presented so as to provide physical insight into lossless operation of the hybrid device.

At the initial light illumination on the PV-TE hybrid circuit, electrical power was generated only from the PV device without any gains from the TE device. However, when a temperature gradient was created in the TE device, thermoelectric voltages were generated, resulting in a net increase in output voltage, as shown in the energy band diagram (Fig. 1c). Since the momentum of the electrons on the hot side was larger than that on the cold side, ΔT led to unequal carrier concentrations on the two sides of the TE device. A potential difference built up due to the momentum unbalance until it became large enough to counteract the net loss of electrons on the hot side, which determined the slope of the Fermi level. The maximum voltage output (*V_TE_*) of the TE device was determined by the difference between the Fermi levels of the PV cell and TE device, as shown in Fig. 1c. Upon absorbing photon energy, electrons were excited and diffused through the cathode of the PV cell, where the maximum voltage output (*V_PV_*) of the PV cell was dictated by the energy gap between the Fermi levels of *n*-Si (E_Fn_) and *p*-Si (E_Fp_). Therefore, the maximum total output voltage in the hybrid cell was *V_PV_* + *V_TE_*. With the reverse polarity operation of the TE device by switching the contact between PV and TE (see Fig. S1), the polarity of the potential difference caused by the photo-generated current (*I_ph_*) (i.e., PV side) was opposite to that caused by the Seebeck effect (i.e., TE side), thereby decreasing *V_oc_* and *I_sc_*in the hybrid circuit.

Figure 1d shows the equivalent electrical circuit of the hybrid operation. The PV cell is regarded as a current source shunted by a diode with a series resistance (*R_S,PV_*) and a shunt resistance (*R_SH,PV_*). The TE device is simply represented by a voltage source accompanying an internal series resistance (*R_i_*). Although a matching current in serially connected electrical systems such as tandem solar cells is governed by a certain subcell generating the lowest current^26^, the overall current in our hybrid circuit is determined by *I_ph_* (i.e., the TE device is a voltage source). In our experimental results, the short circuit current (*I_sc_*) in the hybrid device was independent of ΔT across the underlying TE device. This feature we observed is clearly explained by the following theoretical consideration.

**Figure 2:**
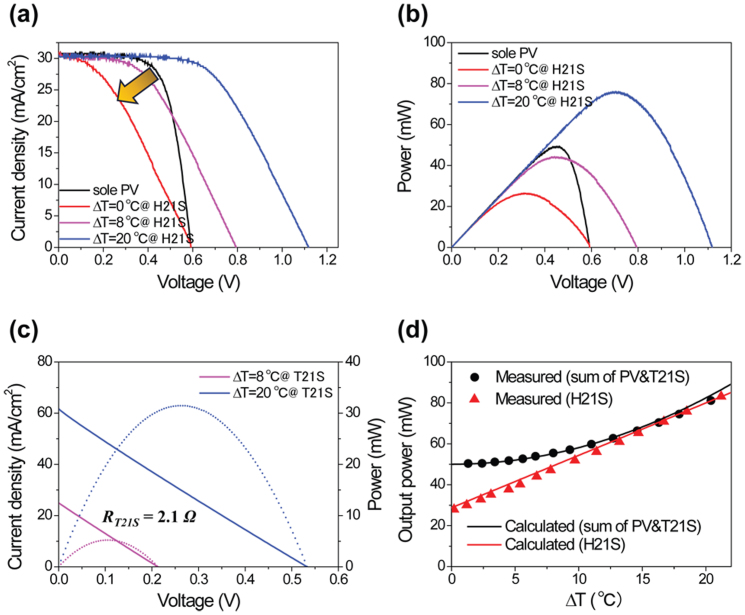
(a) A comparison of the light *J*–*V* characteristics of a PV-T21S hybrid circuit (H21S) under various temperature differences (ΔT). The black solid-curve represents the *J*–*V* characteristic of a sole PV cell. (b) Output power of the sole PV and H21S as a function of voltage. (c) *J*–*V* (solid-line) and output power (dotted-line) curves of a T21S; internal resistance of the TE is 2.1 Ω. (d) The comparison of the measured (symbol) and calculated (solid-line) output power for a simple summation of the PV and T21S (black) and the H21S (red) as a function of the ΔT. (All calculated and measured current values are normalized in terms of effective area).

The *J*–*V* (current density-voltage) and power characteristics of PV, T21S, and their hybrid (H21S) devices are shown in Fig. 2 as a function of ΔT from 0°C to 20°C (see Supplementary for more detailed analysis, Fig. S2). Along with *J_sc_* of 30 mA/cm^2^, H21S yielded *V_oc_* of ~1.12 V at ΔT = 20°C, which is almost the same as the sum of *V_oc_*from PV and TE. *V_oc_* increases with the TE device, in proportion to ΔT as shown in Fig. 2a, however the internal resistance of the TE device often degrades the fill factor (*FF)* of a hybrid circuit. Compared to a sole PV cell, H21S retains a significantly high *V_oc_*, despite a lower *FF*, owing to the additional *V_TE_* induced by ΔT. In order to further improve the overall performance of a hybrid circuit, the reduction in *FF* needs to be as small as possible while increasing *V_oc_* of the hybrid circuit. *J_sc_* is almost unaffected due to the relatively small internal resistance of the TE device.

The output power of the H21S is depicted in Fig. 2b. The maximum output power (*P_max_*) of the hybrid circuit strongly depends on ΔT of the TE device. Note that the internal TE resistance was unchanged although the output power increased with ΔT (Fig. 2c). In order to find the lossless operation condition, we compared the power of the hybrid device with the simple sum of the output values from the individual PV and TE devices (separately operated at their maximum output conditions). Figure 2d is the output power vs. ΔT plot, showing the maximum power (*P_max_*) of 65.2 mW at ΔT = 15°C. When ΔT reaches a threshold value, ~15°C (Fig. 2d), the power output of the hybrid operation equals the sum of maximum power outputs produced separately from the PV and TE devices. This lossless coupling between PV and TE circuits clarified that *I_ph_* determined the overall current under the optimized hybrid PV-TE operation in which the internal TE resistance was low enough to convey *I_ph_*. In addition, *FF* also increased with increasing ΔT as estimated from the positive *dV_oc_*/*dT*. This was consistent with the empirical relationship found in a standard solar cell^28^: 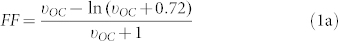

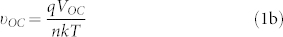
where *q*, *n*, *k*, and *T* stand for electron charge, ideality factor, the Boltzmann constant, and temperature, respectively. These relations clearly indicate that *FF* in the hybrid circuit can be improved by increasing *V_OC_*. In order to gain further insight regarding the behavior of the hybrid circuit, we obtained the power outputs from the individual PV cell and the TE device using equations (2) and (3): 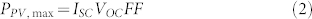

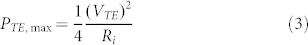


Assuming that PV and TE devices are independently operated, the maximum ideal output power is the sum of those from the two devices, which can be described as: 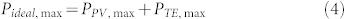
The black solid line in Fig. 2d represents the power output from equation (4). Here, *FF* was 0.703 from the experimental result. *V_TE_* in equation (3) is a thermoelectric voltage generated by ΔT between the hot and cold sides of the TE device. Since the TE device consists of many *p*-*n* couples, *V_TE_* can be expressed by the following equation^29^: 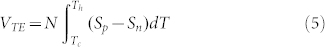
where *N*, *S_p_*, and *S_n_*are the number of *p*-*n* couples, and the Seebeck coefficients of *p*- and *n*-type TE elements, respectively. At temperatures near 300 K, variations in the Seebeck coefficients are negligible. Then, *V_TE_* can be simplified as: 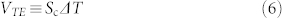
Here, *S_c_* was 0.026 V/K for T21S; 0.012 V/K for T12S and 0.038 V/K for T19L. Note that the load resistances for the PV and TE devices are different at their maximum powers, which necessitates additional apparatuses for optimizing their operation.

Conversely, it is imperative to use a single load resistance (e.g., a battery pack to store electricity) for a hybrid circuit. Depending on the internal resistance and the temperature gradient, the power output of the hybrid device can be dramatically altered. In order to find the optimal operating conditions for maximum power output, we first expressed the output current (*I_pv_*) from the PV cell as follows: 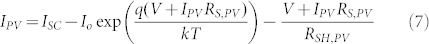
where *I*_0_ and *V* represent a dark saturation current and a load voltage, respectively. According to our measurements, *R_S,PV_*≈ 0.67 *Ω* and *R_SH,PV_* ≈ 300 Ω. In order to find the *I_SC_* and *I*_0_, we fitted the experimental *J*–*V* data (Fig. 2a) from the sole PV cell by adjusting *I_0_*. We found that *I_0_* = 1.26 × 10^−11^ A provided a good agreement with the experimental data. In the hybrid circuit, the current from the PV cell was fed to the TE device, as verified in Fig. 2a (same *I_SC_* irrespective of the presence of the TE device). Therefore, it is reasonable to use the following equation to find the current (*I_hybrid_*) from the hybrid circuit. 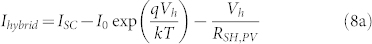

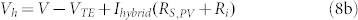


Equation (8) is a non-linear equation, and can be solved only through iterative processes. The power output (red line in Fig. 2d) and power loss of the hybrid circuit can be described as: 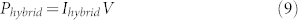

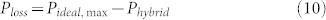


**Figure 3:**
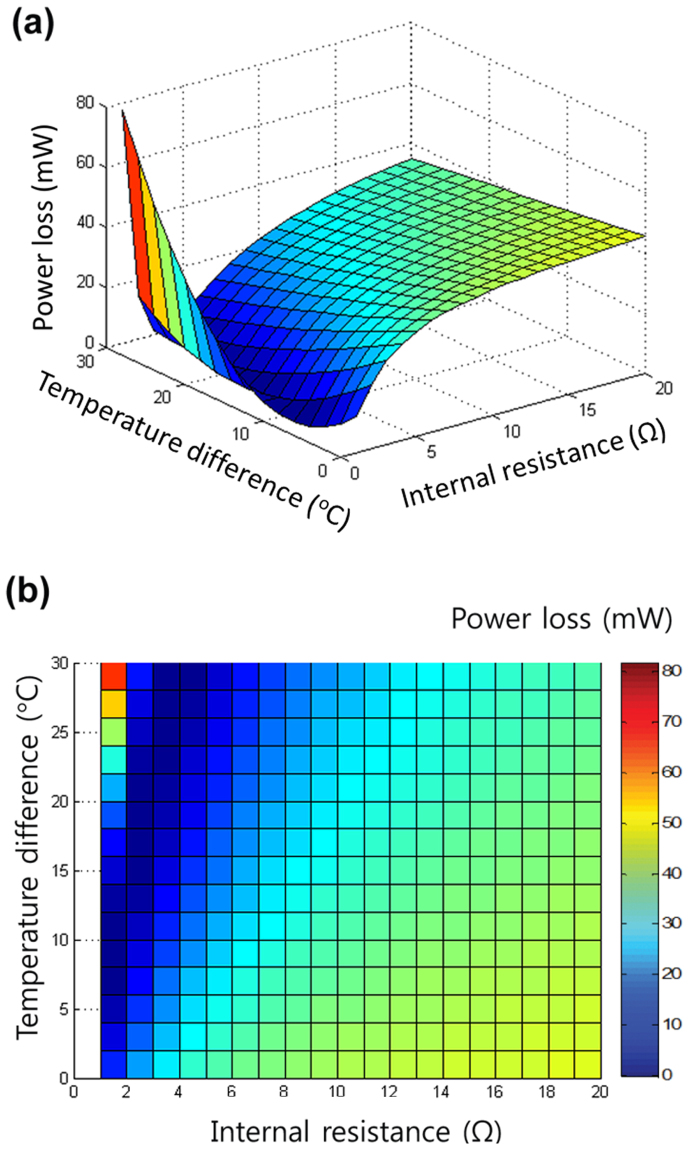
Numerical calculations for lossless coupling. (a) Three-dimensional and (b) plan views of power losses of the hybrid circuit compared to the individual circuit, versus temperature difference (ΔT) and internal resistance of TE (*R_i_*). These graphs indicate the lowest power loss that can be achieved in the range of 1 < *R_i_* < 5 ohm.

The optimal power output can be achieved in the dark blue range shown in Fig. 3, which is determined by the typical trade-off between the increase in *V_OC_* and the decrease in *FF* (Fig. 3).

**Figure 4:**
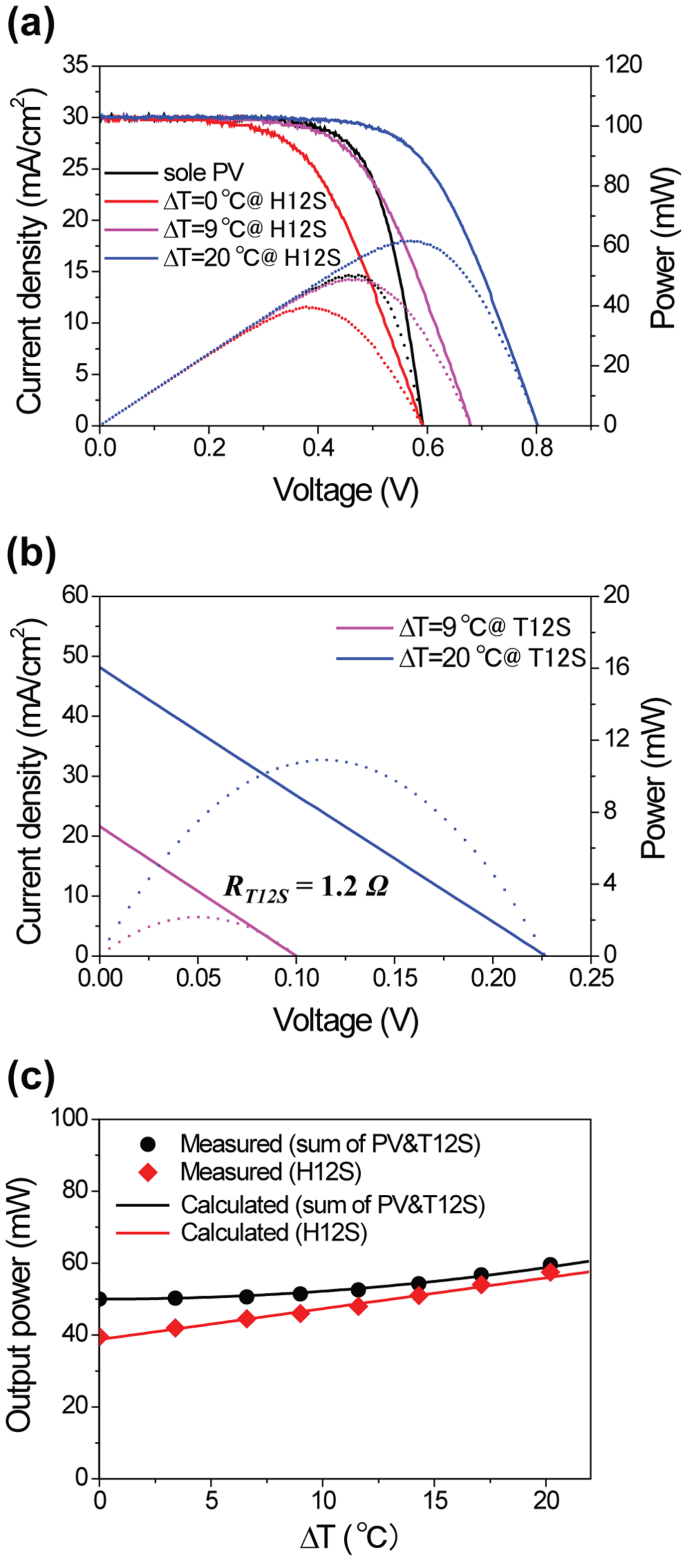
(a) The comparison of the light *J*–*V* characteristics (solid-line) and output power (dotted-line) of a PV-T12S hybrid circuit (H12S) under various ΔT. The black solid-curve represents the *J*–*V* characteristic of the sole PV cell. (b) *J*–*V* (solid-line) and output power (dotted-line) curves of a T12S; internal resistance of TE is 1.2 Ω. (c) The comparison of measured (symbol) and calculated (solid-line) output power for the simple summation of PV and T12S (black) and the H12S (red) as the function of the ΔT. (All calculated and measured current values are normalized in terms of the effective area).

Whereas H21S shows lossless operation, Figure 4 presents the output characteristics with power loss for the PV-T12S hybrid circuit (H12S) along with those of PV and T12S (see Supplementary for more detailed analysis, Fig. S3). Using the same PV cell as H21S, the overall maximum *V_OC_* of H12S increased to 0.81 V at ΔT = 20°C (Fig. 4a). The relatively low *R_i_* reduces the voltage drop caused by the TE internal resistance at the maximum power point in H12S, thereby enabling a higher *FF* compared to H21S. On the contrary, the voltage gain resulted from the *Seebeck effect* for H12S is relatively small due to fewer thermocouple pairs (38 pair legs). The relationships between the number of *p*-*n* couples (*N*) and *R_i_*, or *V_TE_* can be clearly seen in equations (11) and (5), respectively. 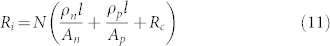
where *ρ* is the electrical resistivity, *R_c_* is the contact resistance, and *A* and *l* are the area and length of the TE elements, respectively. The overall power output of the H12S is lower than the sum of the individual power outputs from the PV cell and T12S, even at ΔT > 20°C, as shown in Fig. 4c. In this case, the reduction in the number of legs, which caused a decrease of TE voltage and an increase of internal electrical conductance, resulted in a net power loss.

**Figure 5:**
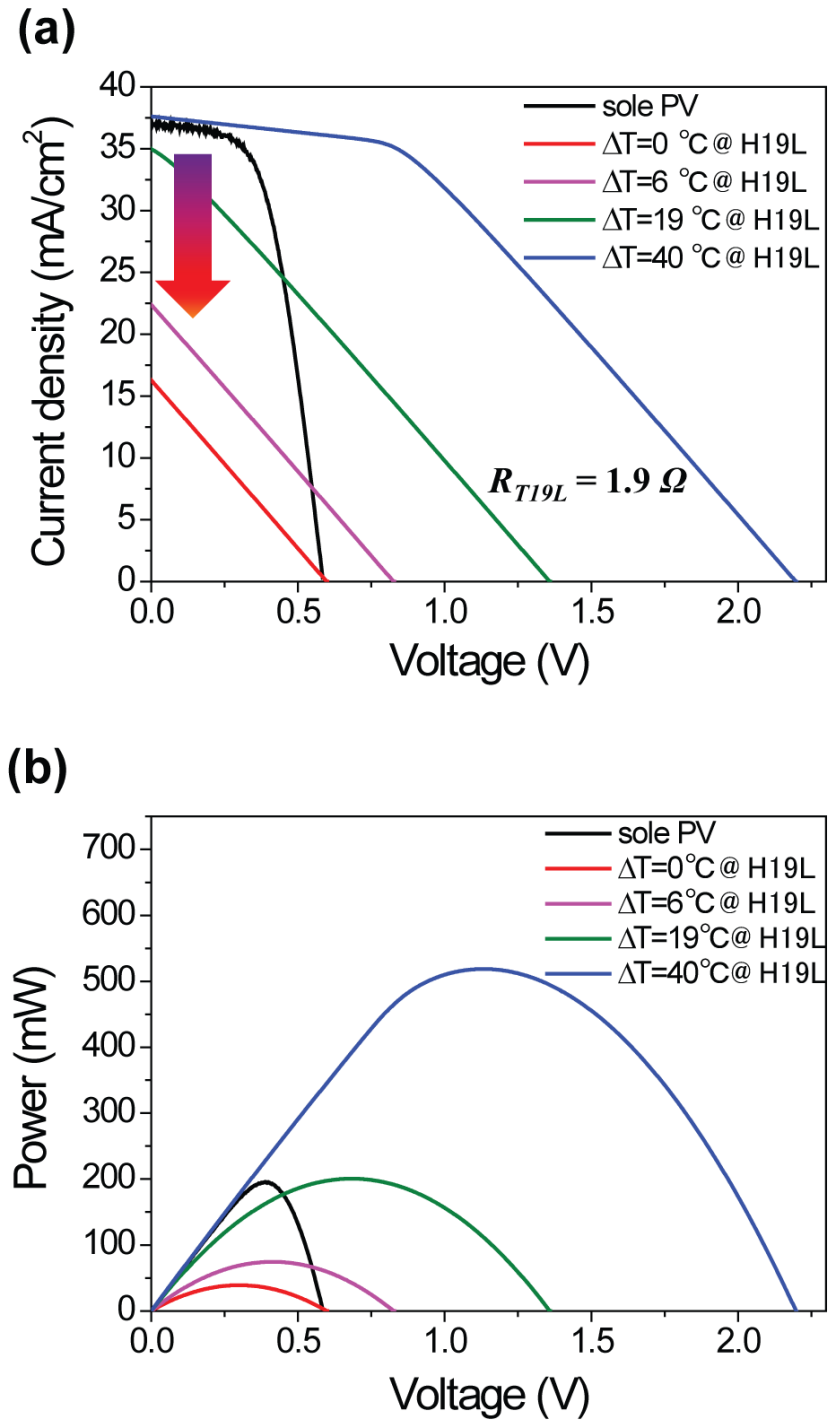
*J*–*V* (a) and output power (b) characteristics of a PV-T19L hybrid circuit (H19L) at various ΔT values across the TE device. The internal resistance of T19L is 1.9 Ω. Lossless matching occurs at ΔT = 40°C with ~250% increase in output power compared to that of a sole PV.

For a H19L device employing a large sized (16 cm^2^) solar cell, the importance of lossless matching becomes more evident (Fig. 5). Because the amount of photo-generated current was four times larger than that of small-sized (4 cm^2^) samples, large reductions in *J_sc_* were shown in Fig. 5a when ΔT across the TE module was less than 20°C. Too large values of *I_hybrid_* (or *R_i_*) cause remarkable increases in the *V_h_*in equation (8b), then iteratively decrease the *I_sc _via* equation (8a), implying a significant power loss by hybridization. For better hybridization, *V_h_* should be decreased through the increase in *V_TE_* in equation (8b), which can be realized by larger ΔT (Fig. 5) as well as the improved TE design (Fig. S4) modifying the length and the number of legs. From Fig. 5b, for instance, ~250% increase of power output is recorded compared to that of a sole PV when ΔT simply reaches 40°C. When the number of legs and the cross-sectional area of each leg for T19L all increased by a factor of 1.5 (which will retain the same *R_i_*), the power output of H19L with the improved design more than doubled, even at a relatively small ΔT = 19°C (Fig. S4a).

## Discussion

Our theoretical and experimental results indicate that resistance matching is of significant importance for optimal operation (i.e. lossless power coupling between a PV cell and a TE device) of a hybrid circuit. Furthermore, we demonstrated that *FF* and voltage gains are obtained by properly selecting the internal resistance and the number of TE elements. It is also important to properly design a TE device in accordance with the *J*–*V* characteristics of selected PV cells. Otherwise, a hybrid PV-TE device may perform worse than a sole PV cell, as demonstrated in our experiments. Our theoretical and experimental study not only showed the feasibility of lossless coupling between PV and TE but also improved the efficiency of the PV device by ~30%, which increased from 12.5% (*P_max_* = 50 mW) to 16.3% (*P_max_* = 65.2 mW) by simply adding a TE device with only a 15°C temperature gradient (see Supplementary Fig. S2 online). This lossless coupling could be achieved in actual solar conditions emulating the variation of incoming solar fluxes (see Fig. S5). We expect that scale-up devices with larger temperature gradients will further improve hybrid device performance. Recent progress in the efficiency of thermoelectric devices may show even better performance. We believe that our simple method could be a practical, viable, and alternative solution, in contrast to further improving the PV cell efficiency by only a few percentage points with costly process integration. Interestingly, during the reverse operation (*Peltier* *effect*) of a TE device, *I_ph_* created an effective heat pump, removing heat from the PV cell. Thus, a TE device can be also used to cool a PV cell in order to prevent PV degradation when *I_ph_* is high enough to surpass the power generation by the *Seebeck effect*. Hybrid operation could be effective for a position-separated operation between PV and TE parts. For instance, PV panels could be installed on a car roof and TE devices on a high-temperature gas exhaust.

## Methods

### Fabrication of a solar cell

A conventional diffused-junction solar cell processing was adopted to fabricate Si solar cells using Czochralski-grown, 230-μm-thick, *p*-type Si(100) wafers (boron doped, 1–10 Ω·cm). The wafer surfaces were textured for antireflection using a KOH/isopropanol solution at 80°C for 20 min. After standard RCA cleaning, the emitter was formed by phosphorus diffusion employing a spin-on-dopant (SOD) method, as described elsewhere^10^. Phosphorous silicate precursors (P509, Filmtronics) were spun onto a wafer, and the *n*^+^-emitter was then formed in a tube furnace using mixed ambient of N_2_ and O_2_ *via* thermal diffusion of gaseous phosphorous at 900°C for 5 min. The residual phosphorus glass that formed by SOD diffusion was removed using a dilute HF solution. A 100-nm-thin, SiN_x_ antireflection layer was deposited on the *n*^+^-emitter using plasma enhanced chemical vapor deposition (Concept 2 Sequel, Novellus), in which the RF power, gas pressure, and a flow rate of source gases (SiH_4_/NH_3_) are 500 W, 2.6 Torr, and 500/4000 sccm at 400°C, respectively. Then, front and back metal contacts were screen-printed using Ag (NS 33-501, Ferro) and Al pastes (AL 53-120, Ferro), respectively. A co-firing step for the electrode formation was performed at 900°C by a rapid thermal annealing system (RTA200H-SP1, New Young M Tech). Two different cell sizes (4 cm^2^ and 16 cm^2^) were prepared.

### Setup of a hybrid cell

The PV-TE hybrid devices for solar energy conversion were fabricated by contacting the backside of a PV cell to a TE cell whose other side was connected to a heat sink. To improve heat conduction, a thermal conductive paste (Sarcon, Fujipoly) with a conductivity of 2.3 W/m·K was used as an adhesive for contact formation between PV and TE devices. PV and TE cells were connected electrically in series, hence a cathode and an anode of a hybrid circuit corresponded to a cathode of a PV and an anode of a TE, respectively. To evaluate the influence of a TE cell on the performance of a hybrid circuit, three different TE cells in which the internal resistances were 2.1, 1.2, and 1.9 Ω, respectively, were adopted for operating each hybrid circuit, labeled also as H21S, H12S, and H19L, respectively. ‘H' and ‘T' stand for ‘hybrid' and ‘TE' cells; ‘S' and ‘L' stand for small (4 cm^2^) and large (16 cm^2^) sized PV and TE cells, respectively. The same size of PV and TE devices was applied in each hybrid operation for precise calculation of conversion efficiencies and electrical powers. For T21S and T19L devices, different structural designs for integrating TE elements were adopted for varying the internal resistances although they had the identical number (127) of legs. For T21S, the cross-sectional area and length of each TE element were 6.4 × 10^−3^ cm^2^ and 0.05 cm, respectively. However, 23.5 × 10^−3^ cm^2^ and 0.12 cm were used for T19L. TE cells used in this study are commercially available from Kryotherm and Laird Technologies.

### Measurement and characterization

As the light and heat source for hybrid operations, a 150 W Xe arc lamp with AM 1.5 G filters was used. The incident flux was measured with a calibrated power meter, also double-checked by the NREL-calibrated solar cell (PV Measurements, Inc.). The temperatures for the PV and TE devices were measured using K-type thermocouples attached onto each device; then, connected to a data logger that recorded one set of reading for every 2 sec. To understand the power generation behavior of the hybrid circuits using various temperature differences, the TE side of the hybrid device was mounted onto a passive heat sink and temperature controller. *I*–*V* characteristics of the PV and hybrid circuit were investigated by using a solar simulator (Peccell technologies) and a potentiostat (Ivium stat, HS-Technologies) under the 1-sun light intensity (100 mW/cm^2^). The system source meter (2636A, Keithely) was used to concomitantly measure *I*–*V* curves of the TE cells. The numerical analysis for hybrid operation successfully estimated the optimal conditions for temperature difference in TE operation as well as structural design of TE elements.

## Author Contributions

K.T.P., S.M.S. and J.H.L. conceived and designed the research. K.T.P., S.M.S., C.Y. and J.H.L. wrote the manuscript. K.T.P. and S.M.S. performed the experiments and analyzed the data. A.S.T. and C.Y. carried out the numerical calculations for analyzing data. H.D.U., J.Y.J., S.W.J., M.W.O., S.D.P. and B.Y. discussed the results and commented on the manuscript.

## Supplementary Material

Supplementary Information Supplementary Information
