# Supplementary material for: Lossless hybridization between photovoltaic and thermoelectric devices
Source: Sci Rep. 2013 Jul 3;3:2123. doi: 10.1038/srep02123 (PMC3699810; doi:10.1038/srep02123)
Supplement: Supplementary Information [file srep02123-s1.pdf]

Supplementary Information

Lossless hybridization between photovoltaic and thermoelectric devices

Kwang-Tae Park[1§], Sun-Mi Shin[1§], Abdullah S. Tazebay[4], Han-Don Um[1], Jin-Young Jung[1], Sang-Won Jee[4], Min-Wook Oh[2], Su-Dong Park[2], Bongyoung Yoo[3], Choongho Yu[4]* and Jung-Ho Lee[1]*

[1] Department of Chemical Engineering, Hanyang University, Ansan, 426-791, Korea.

[2] Creative Electrotechnology Research Center, Korea Electrotechnology Research Institute (KERI), Changwon, 642-120, Korea.

[3] Department of Materials Engineering, Hanyang University, Ansan, 426-791, Korea.

[4] Department of Mechanical Engineering, Texas A&M University, College Station, Texas 77843, United States.

*Correspondence and requests for materials should be addressed to J.-H.L. (jungho@hanyang.ac.kr) or C.Y. (chyu@tamu.edu)

[§]These authors contributed equally to this work.

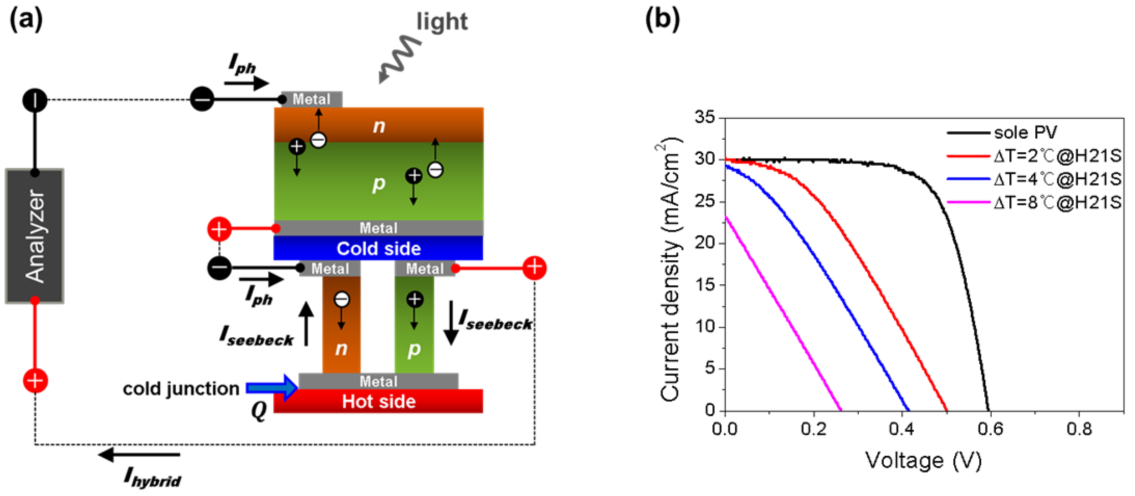

Fig. S1 (a) Schematic illustration of the hybrid circuit when the contact between PV and TE is switched. (b) The comparison of the light *J*–*V* characteristics of the H21S under ΔT. The black solid-curve represents the *J*–*V* characteristic of the sole PV cell. A sign reversion in V_{TE} on the TE output reduces the J_{sc} and V_{oc} of the hybrid circuit.

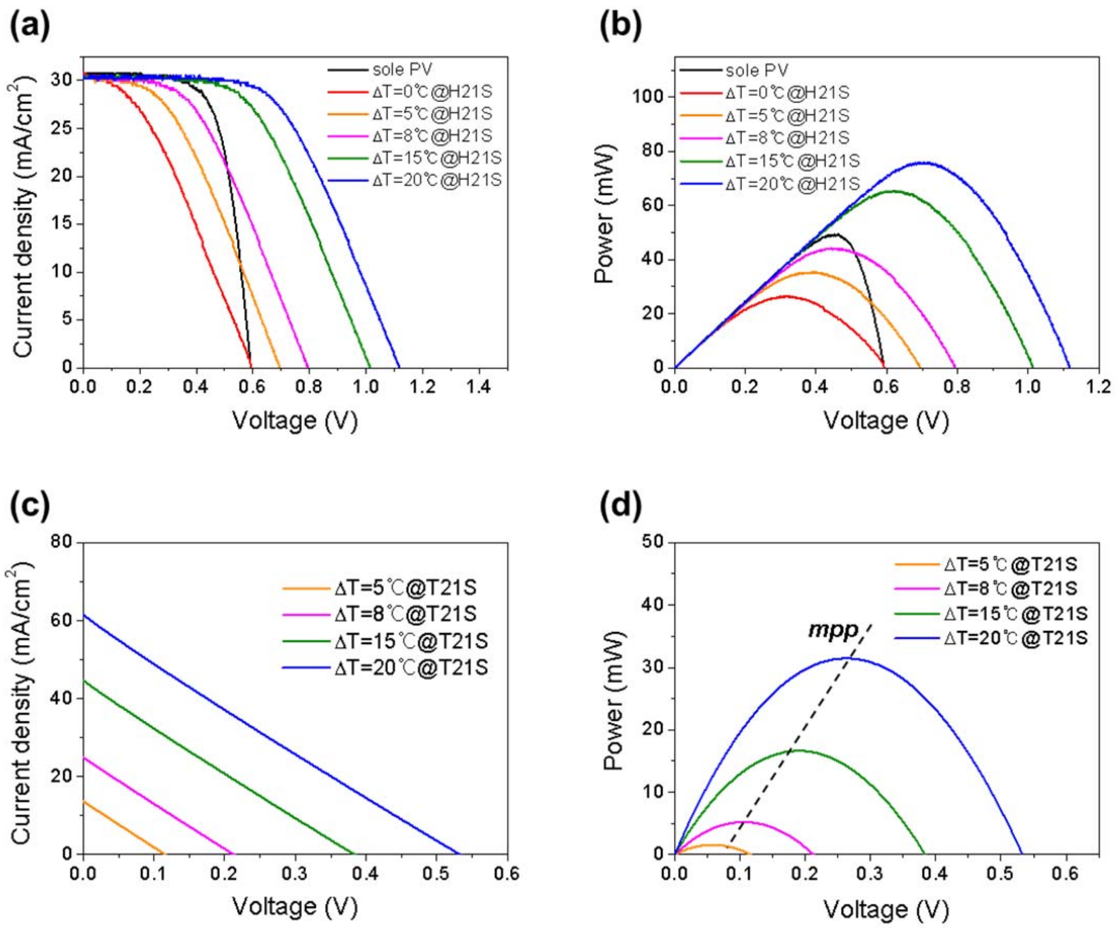

Fig. S2 (a) The comparison of the light *J–V* characteristics of the H21S under various ΔT. (b) Output power of the sole PV and H21S as the function of voltage. The output power curve rises with the increase of ΔT, and each has its maximum at a specific voltage. (c) *J–V* curves of the T21S under various ΔT. (d) Output power curves of the T21S as a function of voltage.

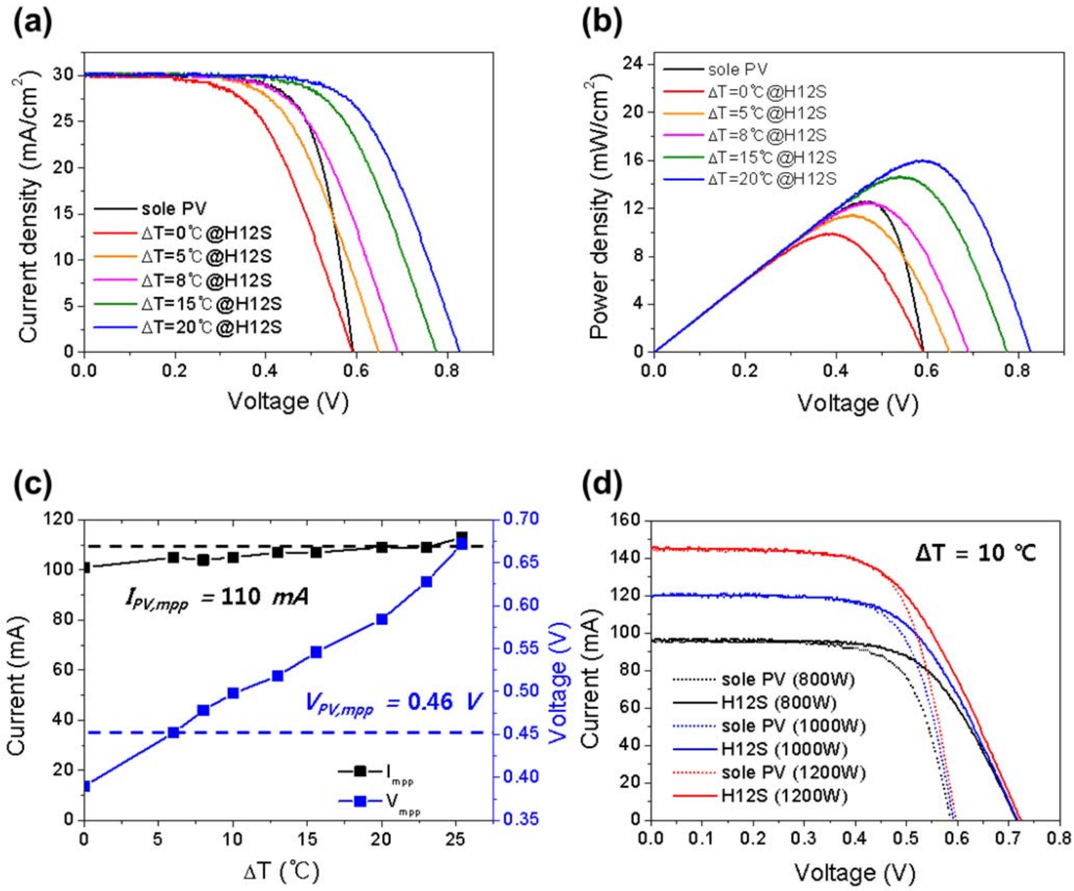

Fig. S3 (a) The comparison of the light *J*–*V* characteristics of the H12S under various ΔT. (b) Output power of the sole PV and H12S as the function of voltage. (c) Dependence of the current at maximum power point I_{mpp} (black) and the voltage at maximum power point V_{mpp} (blue) under the various ΔT. (d) *J*–*V* characteristics of the sole PV (dotted-line) and the H12S (solid-line) under irradiation of different light intensity. The ΔT in H12S is fixed at 10 ºC.

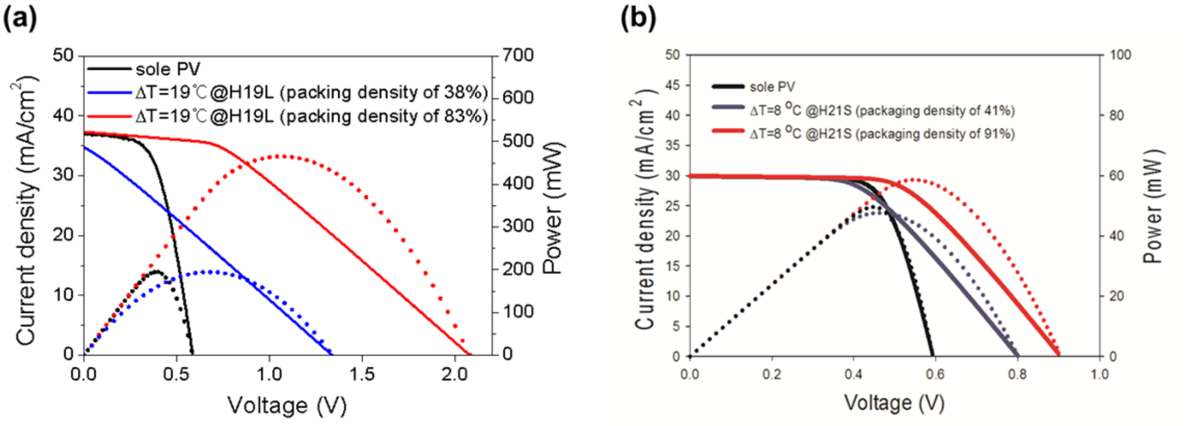

Fig. S4 Calculated *J*–*V* (solid-line) and *P*–*V* (dotted-line) plots for H19L at ΔT=19 °C (a) and H21S at ΔT=8 °C (b). The red lines are for redesigned H19L whose cross-sectional area and number of each leg are all increased by a factor of 1.5. The improved design increases the packing densities of legs to 83% for T19L and 91% for T21S with the same R_i (1.9 Ω for T19L and 2.1 Ω for T21S).

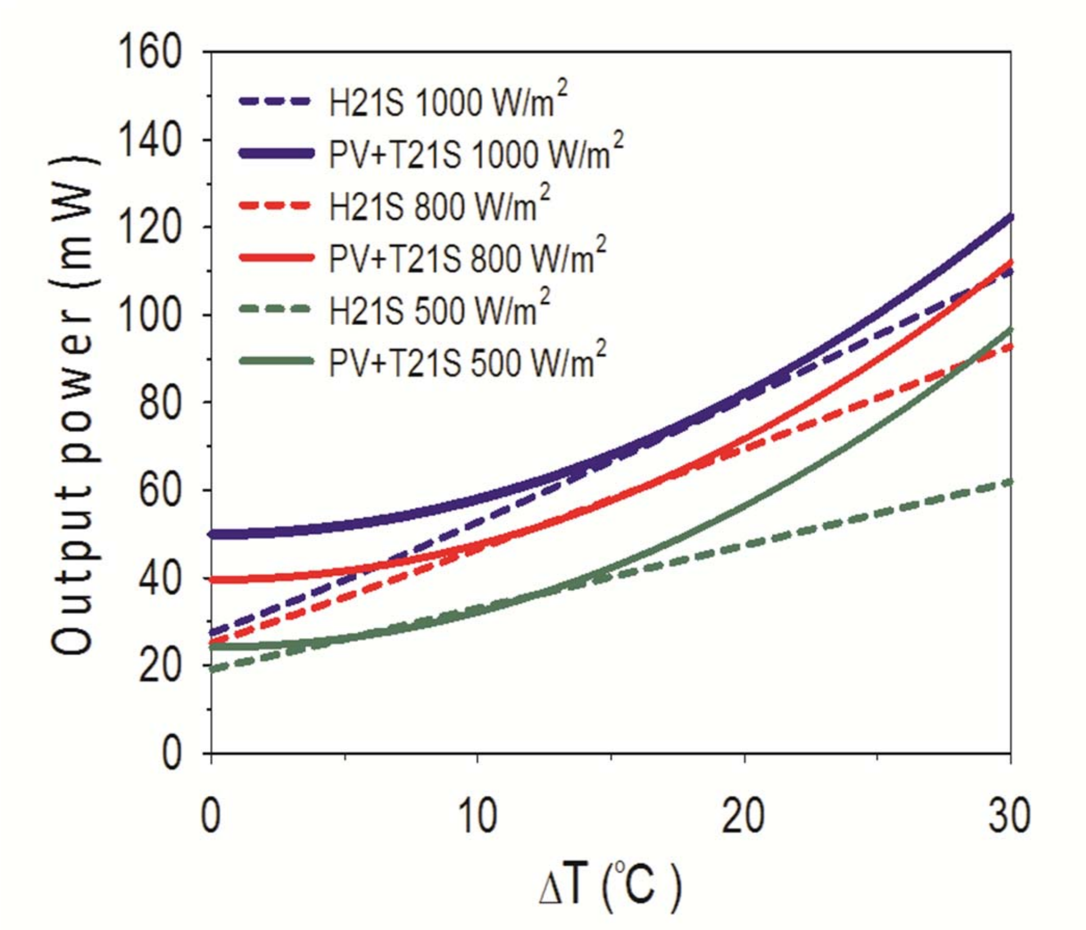

Fig. S5 Calculated output powers for solar irradiances of 1000, 800, 500 W/m^2. Solid-lines are for a simple summation of the PV and T21S, and the dotted-lines are for H21S. The variation of incoming solar fluxes emulates the actual solar conditions.
